# Performance and usability testing of an automated tool for detection of peripheral artery disease using electronic health records

**DOI:** 10.1038/s41598-022-17180-5

**Published:** 2022-08-03

**Authors:** I. Ghanzouri, S. Amal, V. Ho, L. Safarnejad, J. Cabot, C. G. Brown-Johnson, N. Leeper, S. Asch, N. H. Shah, E. G. Ross

**Affiliations:** 1 Division of Vascular Surgery, Department of Surgery, Stanford University School of Medicine, Stanford, CA USA; 2 Department of Medicine, Primary Care and Population Health, Stanford, CA USA; 3 Department of Medicine, Center for Biomedical Informatics Research, Stanford University School of Medicine, 780 Welch Road, CJ350, Stanford, CA 94305 USA

**Keywords:** Data mining, Data processing, Machine learning, Predictive medicine, Peripheral vascular disease

## Abstract

Peripheral artery disease (PAD) is a common cardiovascular disorder that is frequently underdiagnosed, which can lead to poorer outcomes due to lower rates of medical optimization. We aimed to develop an automated tool to identify undiagnosed PAD and evaluate physician acceptance of a dashboard representation of risk assessment. Data were derived from electronic health records (EHR). We developed and compared traditional risk score models to novel machine learning models. For usability testing, primary and specialty care physicians were recruited and interviewed until thematic saturation. Data from 3168 patients with PAD and 16,863 controls were utilized. Results showed a deep learning model that utilized time engineered features outperformed random forest and traditional logistic regression models (average AUCs 0.96, 0.91 and 0.81, respectively), *P* < 0.0001. Of interviewed physicians, 75% were receptive to an EHR-based automated PAD model. Feedback emphasized workflow optimization, including integrating risk assessments directly into the EHR, using dashboard designs that minimize clicks, and providing risk assessments for clinically complex patients. In conclusion, we demonstrate that EHR-based machine learning models can accurately detect risk of PAD and that physicians are receptive to automated risk detection for PAD. Future research aims to prospectively validate model performance and impact on patient outcomes.

## Introduction

Peripheral artery disease (PAD) afflicts 8 to 12 million Americans, incurring up to US$21 billion in annual healthcare costs^1–3^. Current diagnosis entails identifying symptoms ranging from classical claudication to rest pain and non-healing wounds. Unfortunately, only 10–30% of PAD patients report stereotypical symptoms, while clinician and patient awareness of PAD is less than 50%^4,5^. In the absence of unified screening guidelines, PAD remains heavily underdiagnosed. In a screening study of patients greater than 70 years of age, or greater than 59 years with smoking or diabetic history, 55% of PAD patients were undiagnosed^5^. As such, improved methods of PAD detection are needed for timelier risk factor modification and prevention of excess major cardiovascular events, major limb events, and all-cause mortality^6^.

Previously reported PAD risk scores utilizing logistic regression are easily interpretable, but have limited discrimination with area under the curve (AUC) less than 0.8^7–9^. This may be due to reliance on a limited number of demographic, laboratory, and comorbidity data, which may not capture other factors such as lifestyle or biological variables that influence PAD risk and disease trajectory^10^. Electronic health record (EHR) data, in contrast, captures a depth and breadth of information that traditional risk factors do not always capture such as health care utilization (e.g. number of primary care and specialist visits), biological results (e.g. lab values across time), and nuanced factors associated with disease risk (e.g. mental health). Given the large number of data points and non-linear associations, machine learning algorithms applied to EHR data may improve performance of PAD risk detection models.

A further issue in developing risk models is clinical adoption. While clinical risk scores to enhance PAD detection exist, evidence of routine usage to inform PAD screening is lacking and impediments to using risk scores may be limited by lack of implementation considerations. Usability testing serves the dual purpose of bringing physician stakeholders into the process of designing risk models that can improve adoption through user-centered design^11^. “Think aloud” protocols in which subjects are encouraged to verbalize mental processes while performing a task have been used extensively in designing clinical decision support interventions^12,13^. In particular Think aloud enables investigators to identify the areas of an interface that draw users’ attention and how these features influence cognitive processing^14^.

Our hypotheses are that machine learning models for PAD classification using EHR data can improve accuracy of PAD detection and usability testing can help inform development of a risk prediction tool to increase physician usage and engagement. In this paper we evaluate the performance of traditional risk factor models versus machine learned models in classification of PAD using both classical machine learning and deep learning algorithms and electronic health record data. We integrate our best-performing model into an interactive dashboard for Think aloud usability testing with primary care and cardiovascular specialty physicians in order to inform implementation efforts.

## Methods

### Data source

The Stanford Institutional Review Board approved this study. We received a waiver for informed consent because the research was deemed minimal risk to participants, the research could not practicably be carried out without the waiver, no identifiable information was used and the waiver did not adversely affect the rights and welfare of subjects. All methods were performed in accordance to the Helsinki Ethical Principles for Medical Research Involving Human Subjects. Data were derived from the STAnford Medicine Research Data Repository (STARR). Data include de-identified EHR clinical practice data at Stanford from 1998 to 2020 featuring over 4 million adult patients, $>$ 75 million visits, $>$ 65 million notes, $>$ 67 million procedures, $>$ 350 million labs and $>$ 55 million prescriptions. These data were converted to the Observational Medical Outcomes Partnership common data model (OMOP CDM)^15,16^. Described elsewhere, in short, the OMOP CDM enables the use of standardized definitions for different data elements within the EHR^17^. This enables better reproducibility across care sites and portability of code to other institutions that utilize the OMOP CDM for their EHR data.

### Cohort

We aimed to develop models to identify cases of PAD prior to the diagnosis date to mimic the scenario of identifying disease prior to clinician diagnosis. To do this, we defined PAD as those with at least two separate ICD-9/ICD-10 or CPT codes and/or PAD mentions in their notes and no exclusion codes (Supplemental Table 1). Only data collected 60 days prior to their diagnosis was included to ensure codes associated with PAD diagnosis were not used in our models. Controls were defined as those without any codes or text mentions for PAD in their health records. Patients were excluded if they had $<1$ year of data. Age was calculated based on the patient’s age at the time of their last included visit. Our final models included all adult patients 50 years and older with at least 1 year of EHR data.

### Traditional risk score model

To evaluate the performance of traditional risk score models, we recapitulated the model developed by Duval and colleagues to estimate risk of prevalent PAD using EHR data instead of registry data^8^. To do this we defined risk factors for PAD as outlined in their model, which included hypertension, hyperlipidemia, diabetes, coronary artery disease (CAD), cerebrovascular disease, congestive heart failure, and BMI. Patients had to have at least 2 affirmative codes/note mentions in their record to be classified as having a specific comorbidity. Because we did not have many blood pressure measurements for each patient, we modified the Duval hypertension definition by only including whether or not a patient was diagnosed with hypertension without the degree of hypertension (e.g. Stage I or II). Our EHR-based definitions for different risk factors are detailed in Supplemental Table 2. BMI was calculated based on the patient’s average BMI in their health record after excluding outlier values. Race/ethnicity was derived from the EHR and coded as Caucasian, Asian, Black, or Hispanic. If these data were missing or not one of the aforementioned categories, the Race/ethnicity variable was coded as “Other” to align with the Duval model categories. Observations with other missing data (e.g. BMI) were found to be infrequent and were dropped from the modeling process. Finally, to calculate individual risk scores for PAD we employed 2 approaches—calculating their nomogram score (and evaluating the overall C-statistic achieved from this score) and using the risk factors in a logistic regression model which was trained on 75% of data and then tested on 25% of the data using fivefold inner and outer cross validation. To mimic an expected 10% prevalence of PAD in a cohort of patients $\ge$ 50 years of age, the training, testing, and each of the fivefold outer cross-validation sets had a 1:10 case/control ratio.

### Machine learning model

We built machine learning models using EHR data formatted in the OMOP CDM. Specifically, we used the same cohort of PAD cases and controls, but instead of traditional risk factors we extracted all of an individual’s EHR data (ICD-9/10, CPT, labs, medications, and observation concept codes) from the date of entry into the health care system to 60 days prior to PAD diagnosis (for cases) or prior to the last visit date (controls). This was done to mimic a use case in which a patient’s risk of PAD would be calculated prior to a definitive diagnosis. We maintained a sparse matrix and did not impute missing values in order to maintain a real-world representation of EHR data and because there is no consensus on how best to model missing EHR data, where data may be missing completely at random or for reasons related to disease phenotypes, which can be useful both clinically and in training machine learning models^18^. Using least absolute shrinkage and selection operator (LASSO) and random forest algorithms, we used 75% of patient data for model building and 25% for model testing. We performed fivefold inner and outer cross-validation. A 10% prevalence of PAD (1:10 case/control ratio) was designed into the training set, testing set, and each outer fold. The best model was chosen based on the AUC.

### Deep learning model

**Figure 1:**
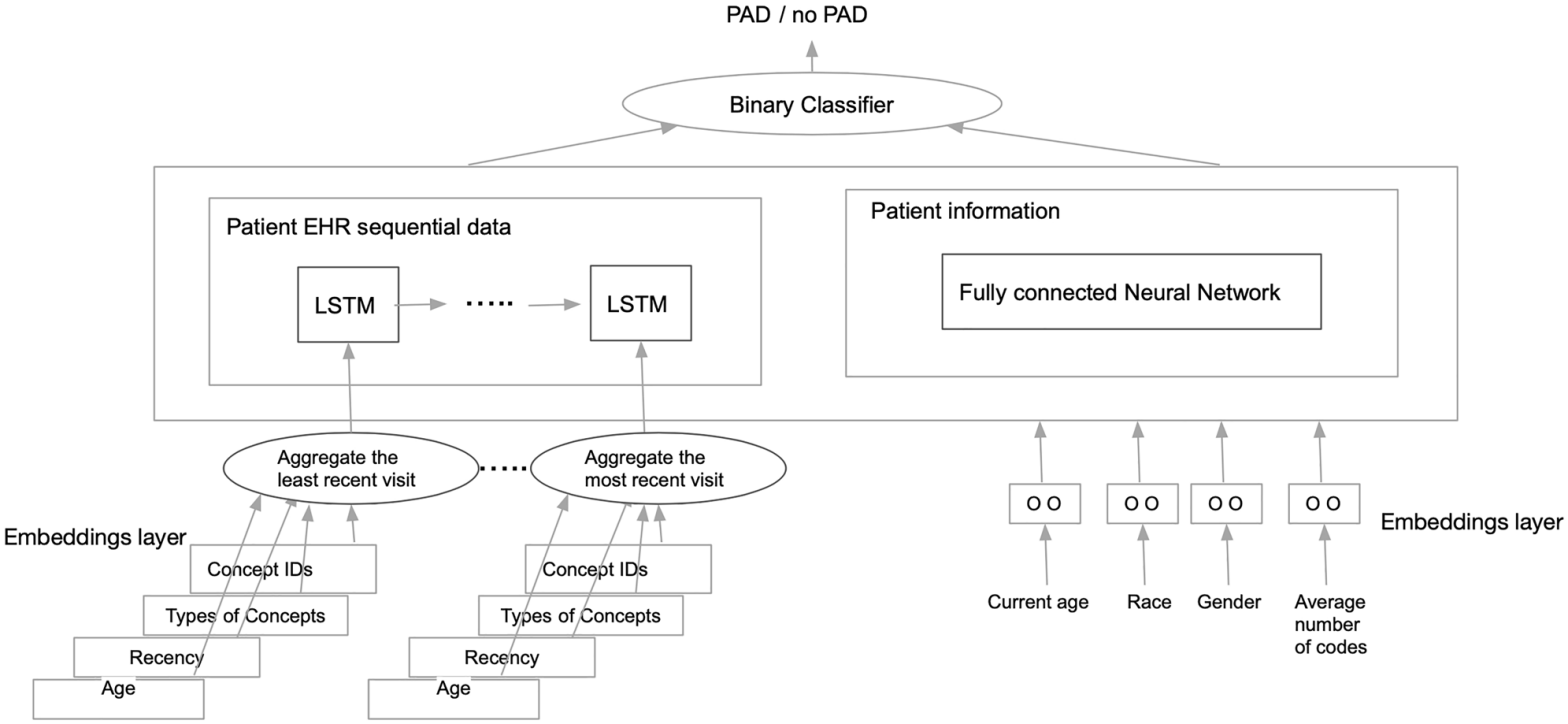
Sequential clinical data and aggregated demographic data are modelled in parallel then combined to make a final classification of PAD versus no PAD in deep learning model.

Disease progression is a dynamic phenomenon. Therefore, algorithms that can take a patient’s health care journey through time into account may produce more accurate estimates of disease risk. We developed a deep learning architecture based on a Recurrent Neural Network (RNN) algorithm using a variation known as Long Short-Term Memory (LSTM)^19^. Though classic RNN algorithms aim to capture time series data of an arbitrary length (i.e. include patient data over short or long periods of time), in practice, the longer the time-series horizon is, the more likely these algorithms are to lose predictive power as they “forget” or over-emphasize certain model features that occurred much earlier in the time series. To overcome this problem, we used the LSTM variation to model time-series data. We built a deep learning model with two components—a fully-connected neural network that modelled static demographic features (Fig. 1) and an LSTM component that modelled sequential EHR data.

To build our deep learning model we used the same EHR data used in our machine learning model described above and included time stamps from PAD diagnosis or last recorded visit (for controls). We also added a dimension known as “recency” that captured how close to PAD diagnosis or end of patient record (for controls) a diagnosis or lab value, for example, appeared in the record. The recency variable ranged from 1 to 10. We used a Keras framework for each of the layers in our architecture. As illustrated in Fig. 1, for the LSTM model, we first started with an embeddings layer that took in data from each clinical visit (age, concept codes, etc.) and produced a new latent dimension. We then aggregated these data, which served as input to the LSTM model. In parallel, we used a fully connected neural network to aggregate age, race, gender, and total number of features. We then aggregated these two components as input to a binary classification layer that classified the patient as having PAD or not.

### Statistical analysis

We used chi-square tests and Student’s t-tests to compare demographic and clinical factors in our patient cohort. We used the De Long Test of significance to compare AUCs^20^. Model calibration was evaluated using visual analysis of calibration curves. We used R software (version 3.6.3)^21^ and Python (version 3.7.10) ^22^ for model building, evaluation and statistical analysis.

### Usability testing

Best-performing model output, mock patient demographics, selected clinical features, and current guidelines on treatment of patients with PAD were incorporated into two clinician-facing dashboards. The “Tabbed Dashboard” stored patient demographics, visit summaries, and risk factors in embedded links, while the “Unified Dashboard” displayed this information in one page. A prediction score was generated as a percentage probability of the patient having PAD based on normalized data. The dashboard recommended screening for values greater than 50%.

Physicians specializing in primary care, cardiology, or vascular medicine were recruited via email, and qualitative usability testing was performed with 25-min semi-structured interviews. Participants were asked to describe their approach to diagnosing PAD prior to listening to a patient vignette and navigating the dashboards in randomized order. A facilitator encouraged participants to think aloud using prompts previously described by Virzi et al.^23^. Participants were recruited until thematic saturation, and transcripts were analyzed thematically.

## Results

### Cohort characteristics

We identified 3,168 patients with PAD and 16,863 controls all aged 50 years and older. Table 1 details comparisons across PAD cases and controls. Amongst PAD cases, 60% were male, while 45% of controls were male. Approximately $70\mathrm{\%}$ of our entire cohort were Caucasian. As expected, those with PAD had a higher burden of comorbidities with 44% of PAD cases with history of cerebrovascular disease (CVD) and 72% with coronary artery disease (CAD). Heart failure (HF), hypertension (HTN), diabetes and hyperlipidemia (HLD) also occurred more frequently amongst PAD cases compared to controls.

**Table 1:** Descriptive statistics of case and control cohorts.

	Cases (N = 3,168)	Controls (N = 16,863)	*P-*value
**Patients**			
Age (mean, y ± SD)	74.8 (± 11)	67.3 (± 11)	2e-16
Number (%) of Females	1386 (40)	9202 (54.6)	$<$ 2e-16
**Race/ethnicity, %**			
Caucasians	69.2	70.4	NS
Black	5.8	4.1	2.5e-05
Asians	14.1	14.1	NS
Hispanic	9.4	9.6	NS
Other	1.5	1.8	NS
**ASCVD, %**			
CVD (%)	44.3	17.1	$<$ 2e-16
CAD (%)	71.7	36.1	$<$ 2e-16
HF (%)	46.4	20.2	$<$ 2e-16
HTN (%)	85.6	54.4	$<$ 2e-16
Diabetes (%)	21.7	8.4	$<$ 2e-16
HLD (%)	39.4	23.5	$<$ 2e-16
BMI (mean, y ± SD)	27.7 (± 6)	27.6 (± 6)	NS
Current smokers (%)	14.1	13.6	0.003

ASCVD—Atherosclerotic cardiovascular disease; CVD—cerebrovascular disease; CAD—coronary artery disease; HF—heart failure; HTN—hypertension; HLD—hyperlipidemia; BMI—body mass index.

### Results of traditional risk score model

**Figure 2:**
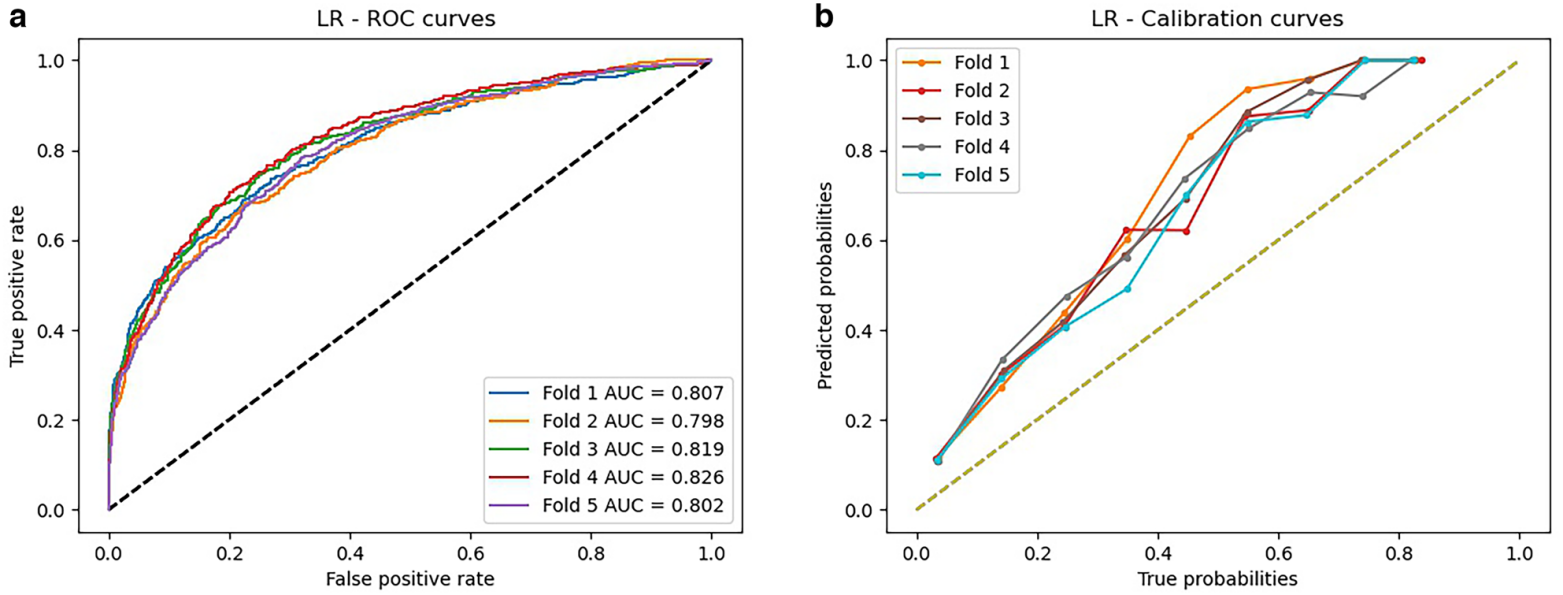
Logistic regression (**a**) receiver operating characteristic curve and (**b**) calibration curves for five outer validation folds. AUC—area under the curve.

Three variables had missingness: race (1.8% of cohort), body mass index (BMI, 0.06%), and sex (0.03%). Applying two different approaches to our traditional risk factor model, we calculated performance using a logistic regression model based on factors outlined by Duval and colleagues^8^ in addition to calculating a nomogram score as recommended by the authors. The nomogram score, unlike the logistic regression model, can be hand-calculated by physicians. The logistic regression model achieved an average AUC of 0.81 (Table 2), with the highest AUC achieved of 0.83 (Fig. 2A,B, Supplemental Table 3). The nomogram model achieved an average AUC of 0.64, with the highest AUC being 0.66 (Supplemental Table 4). Overall, our calibration curve demonstrates that the logistic regression model tended to overestimate risk across low and high-risk individuals.

**Table 2:** Model results comparison.

Model	Average AUC	Average specificity	Average sensitivity
Logistic regression	0.81	0.73	0.75
Nomogram	0.64	0.62	0.62
Machine learning	0.91	0.81	0.83
Deep learning	0.96	0.88	0.95

The average area under the curve, sensitivity, and specificity for each model. AUC—area under the curve.

### Results of machine learning model

**Figure 3:**
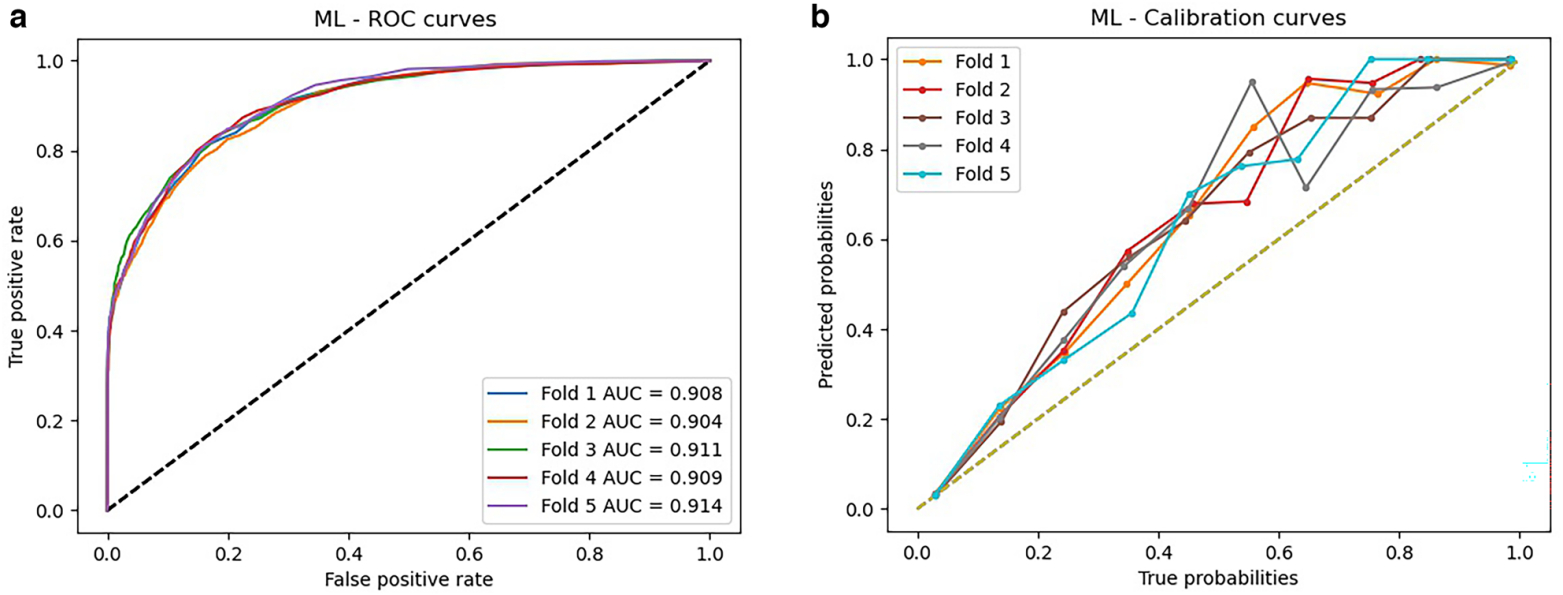
Random forest (**a**) area under the curve and (**b**) calibration curves for five outer validation folds. AUC—area under the curve.

**Figure 4:**
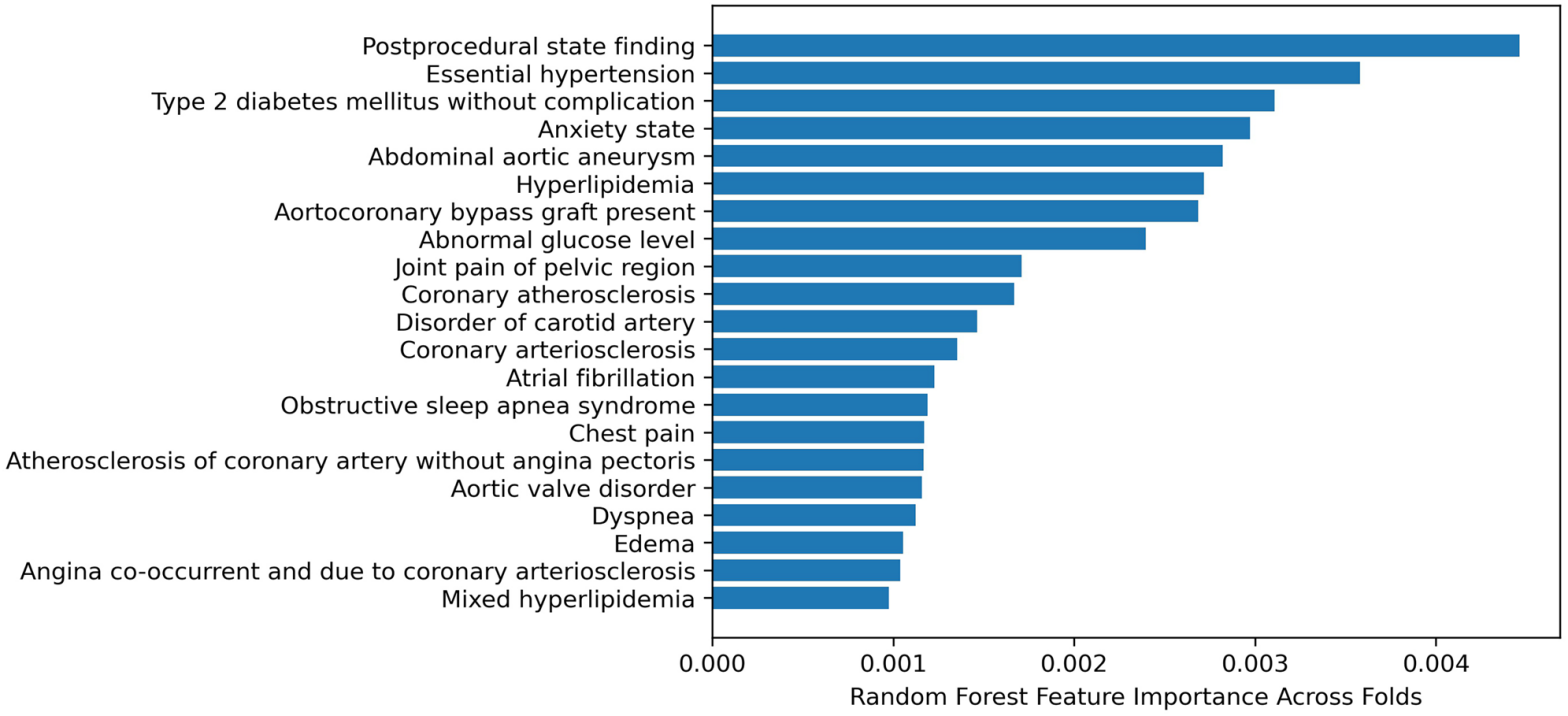
Features most heavily weighted in discriminating between cases and controls in random forest model, based on feature importance averaged across folds.

Our best performing machine learning model used a random forest (RF) algorithm and achieved an average AUC of 0.91 (Table 2, Supplemental Table 5). Compared to the logistic regression model, the RF model had a significantly higher AUC (*P* < 0.0001) and similar calibration characteristics (Fig. 3a, b). Feature importance was calculated as the average across all five outer validation folds and the features most heavily weighted in the RF model are illustrated in Fig. 4. As expected, model features were enriched for co-morbid cardiac and vascular diseases.

### Results of deep learning model

**Figure 5:**
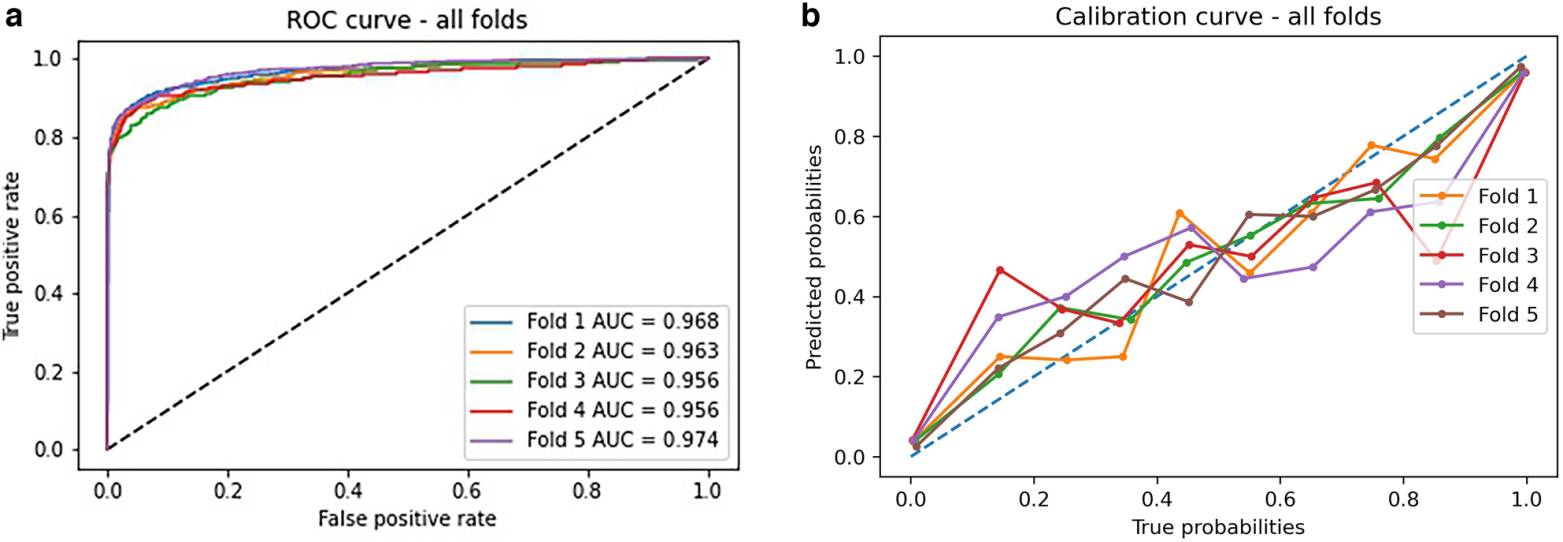
Deep learning model (**a**) area under the curve and (**b**) calibration curves for five outer validation folds. AUC—area under the curve.

Our deep learning model achieved the best AUC results (Table 2, Fig. 5a, b, Supplemental Table 6), with significant improvements in AUC compared to the logistic regression and random forest models (*P* < 0.0001). Calibration curves demonstrate more variability across folds, but overall better calibration across high and low risk groups compared to the random forest and logistic regression models.

In comparing the results of each model, we identified how many more true positive cases were found with increasing model sophistication (Table 3). Compared to the logistic regression model, the random forest and deep learning models produced nearly 10% and nearly 25% increases in identification of true positive cases, respectively. The deep learning model increased identification of true positive cases by nearly 14% compared to the random forest model.

**Table 3:** Percentage increase in true positive cases by increasing model sophistication.

% increase of true positives	Random forest	Deep learning
Logistic regression	+ 9.6%	+ 24.6%
Random forest	–	+ 13.7%

### Usability testing

**Figure 6:**
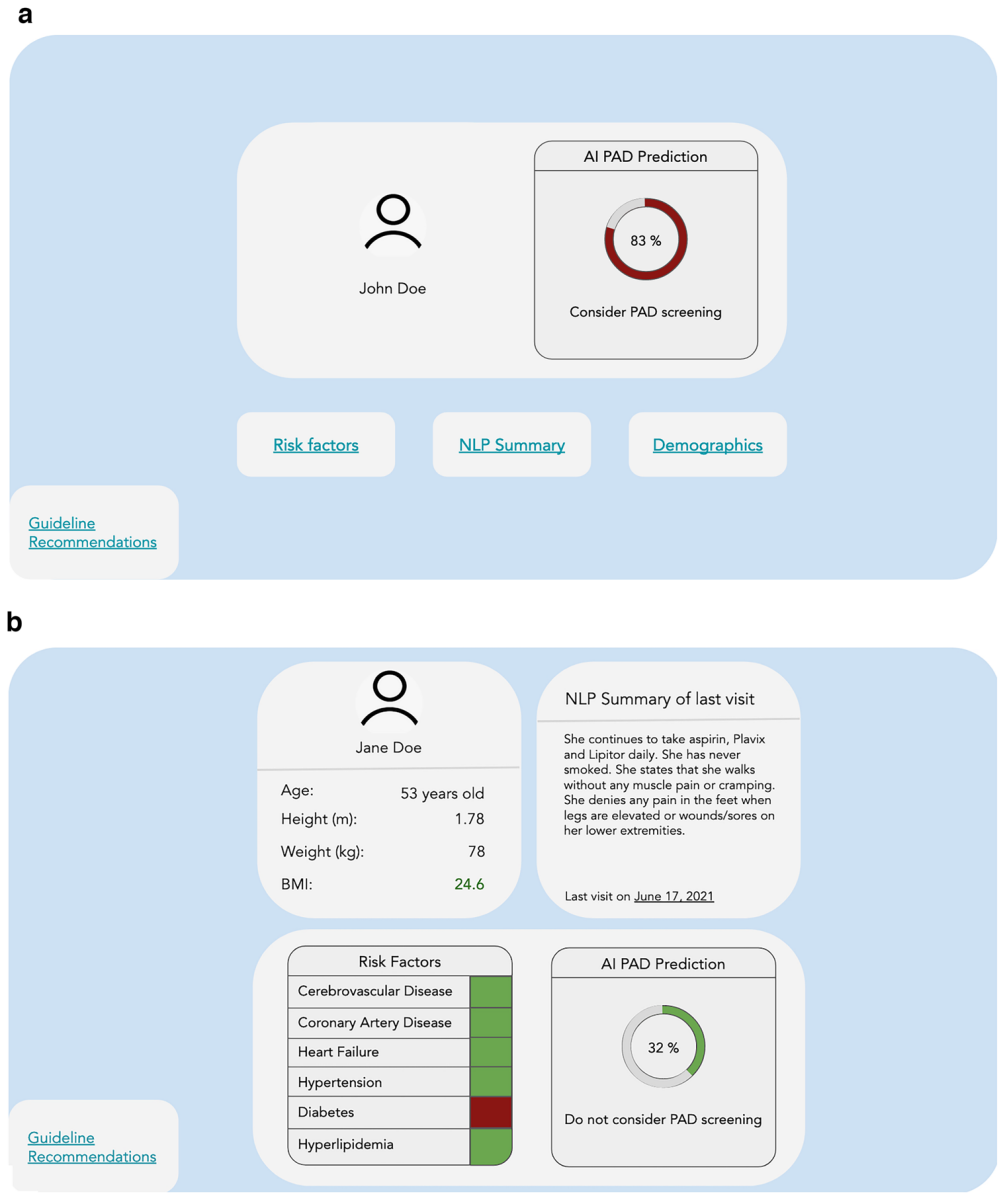
Dashboards for presentation of patient risk of peripheral artery disease. (**a**) Tabbed dashboard. Further information on risk factors, patient summary, demographics and guideline recommendations are available only through directly clicking labeled links. (**b**) Unified Dashboard. All patient information is displayed in one frame of reference with guideline recommendations made available through clicking a link. AI—artificial intelligence; NLP—natural language processing; PAD—peripheral artery disease.

Dashboard designs we developed for user testing are illustrated in Fig. 6a, b. Twelve clinicians (6 primary care physicians and 6 cardiovascular specialists) underwent usability testing using these dashboards. From interviews, three themes emerged: ease of understanding, ease of use, and acceptability (Table 4).

**Table 4:** Usability themes and subthemes.

Themes	Subthemes	Example Quote
Ease of understanding	Difficulty interpreting prediction output (6 of 12 participants)	“The prediction score … I don't know how to contextualize that. Is 75% to 100% when we consider screening? Some kind of scale would be helpful.” (Participant 7)
Ease of use	Desire for electronic health record integration (7 of 12 participants)	“I don't know if I would necessarily click on a link to go to another website. It is just one step removed that may decrease compliance.” (Participant 5)
	Reducing clicks while navigating (8 of 12 participants)	“It took me three clicks to get here; it would have been better if it was simpler. It does affect my usage and efficiency and maybe satisfaction.” (Participant 2)
Acceptability	Improving peripheral artery disease diagnosis (9 of 12 participants)	“[I]t would help remind me, especially for more complicated … it can be difficult… when you're just kind of inundated with a lot of different problems in a single 30-min visit … to continue to have this on your radar (Participant 11)”
		“If you have somebody … on the borderline, … it would be nice to see whether you know your overall gestalt matches with the gestalt of the of the computer… If it was like 83% … I would maybe order that test.” (Participant 9)
	Low priority in diagnosing peripheral artery disease amongst primary care physicians (4 of 12 participants)	If I'm trying to do other screenings, I have to put that risk benefit ratio in the context of everything else. If they haven't had their colonoscopy or mammogram, do I send them for that if they have limited bandwidth? Or do I send them for a PAD screen? (Participant 4)
		“[Peripheral artery disease] … it's not like coronary disease, where if you miss it, somebody is going to have an acute event and … death could ensue, right? Whereas if you have peripheral vascular disease that you haven't picked up and they're not symptomatic …, is it really going to make a big difference?” (Participant 1)
	Varying perceptions of machine learning (2 of 12 participants)	“I'm for machine learning to help me be a better doctor. It's going to help me not miss diseases, and it's going to help me manage diseases better.” (Participant 4)
		“I think it's hard to know what to make of any particular AI prediction… I …would like to have a link to maybe a paper that was peer reviewed saying … this works” (Participant 11)

#### Ease of understanding

Half of providers indicated that the value displayed by the prediction model was difficult to interpret. Some inquired about the numerical threshold at which the model recommended screening (25%), while others wondered whether the value represented a positive predictive value or other measure of certainty (33%).

#### Ease of use

Physicians unanimously preferred a Unified Dashboard (100%) (Fig. 6b), with the majority emphasizing the importance of reducing the number of clicks required to visualize information (67%). Since the order of dashboards presented to participants was randomized, the preference for the Unified Dashboard was not influenced by primacy bias. Most physicians preferred integration into the health record, specifically recommending a link within the EHR interface or direct import into patient notes (58%). Some stated that in the context of a busy clinic, it would be difficult to integrate an external website into their workflow.

#### Acceptability

The majority of practitioners generally felt that the dashboard could improve their ability to diagnose PAD (75%), particularly when dealing with complex patients or diagnostic uncertainty. However, acceptability amongst primary care providers was influenced by perceptions that a missed diagnosis of PAD was less urgent compared to other screening initiatives (67% of primary physicians). Participants unanimously reported that they had never implemented a machine learning-based tool into their clinical workflow (100%). While the majority of participants had positive perceptions of machine learning (83%), two dissenting opinions highlighted distrust due to the lack of algorithmic transparency. One physician cited concerns regarding the lack of clarity in how patient factors influenced the model but suggested that adding information regarding decision threshold could address this. Another participant stated that their own unfamiliarity with machine learning was a personal barrier to acceptance, although relevant publication in a peer-reviewed journal would be beneficial.

## Discussion

In this work we demonstrate the feasibility of using machine learning and deep learning algorithms for detecting patients at high risk of having PAD prior to diagnosis using EHR data. We found that deep learning models, which incorporated timing of diagnoses, procedures, medications and other EHR data performed significantly better than a traditional risk factor model and standard machine learning approaches. Furthermore, we found that “Think aloud” stakeholder interviews enabled greater insight into developing an implementation strategy that may be more appealing for busy clinicians. Clinician stakeholders evaluating a model dashboard felt model implementation could improve diagnosis for complex patients or those with moderate pre-test concern for PAD, and recommended EHR integration and click reduction to facilitate adoption.

We have previously shown that machine learning-based models can identify previously undiagnosed PAD patients using clinical trial data^24–27^. In work by Ross and colleagues, data from the Genetics of Peripheral Artery Disease (GenePAD) study was used to build traditional and machine learning models. By applying a systematic comparison, Ross et al. showed that machine learning-based models outperformed logistic regression models for identification of PAD patients and estimating risk of mortality^24^. In our current work, we extend those findings and show that it is possible to develop accurate machine learning models using EHR data. This is an important advance as EHR data can often be missing, sparse, and unstructured, and therefore, making typical linear models less likely to perform well.

Others have described different methodologies for identifying PAD using EHR data. For example, Afzal and colleagues applied natural language processing (NLP) to automate detection of prevalent PAD in the EHR^28^. In their work, Afzal et al. extracted several key concepts describing PAD and used them to develop rules to classify patients as having PAD or not having PAD. In comparison with their previously proposed method that used ICD-9 diagnostic codes and also a combination of ICD-9 codes with procedural codes to identify PAD patients^29^, they demonstrated that NLP methods can achieve good accuracy (NLP: 91.8%, full model: 81.8%, simple model: 83%). Although novel, Afzal and colleagues' work focused on identifying already diagnosed PAD cases while our model aimed to identify PAD prior to patient diagnosis. Moreover, EHR data are notorious for being highly unstructured. Therefore, developing a comprehensive set of rules to capture all variations and combinations of concepts describing a clinical event could be a cumbersome task, and thus algorithms that can automate data extraction and use of relevant data are important.

Our deep learning model outperformed both our traditional and machine learning models. We believe this is the case for a few reasons. While traditional risk factor and machine learning algorithms tend to use aggregate patient data to make predictions, deep learning algorithms such as recurrent neural networks can take timing of feature occurrence into account when making predictions. Furthermore, certain features such as medications, diagnoses and procedures that occur at certain time points in a patient’s history may be especially predictive of an outcome and these relationships can be modelled in deep learning architectures. Furthermore, by utilizing an additional modeling layer known as Long Short-Term Memory (LSTM) in our deep learning architecture we were able to model data from longer time horizons^30^. Lastly, deep learning models, through the complexity enabled by multiple neural network layers, enable modeling of more complex non-linear relationships compared with traditional machine learning algorithms. Though it is sometimes argued that deep learning models may be too complex and not practical for point of care use^31^ we found that it took an average of 1.25 h to train our deep learning model and 2.6 ms to make a prediction for a single patient. Thus, models can be potentially retrained on a weekly or monthly basis to ensure models have up-to-date data while point of care predictions can be made even during a patient’s clinic visit when clinicians may have anywhere from 15 to 30 min to see a patient.

While model performance is an important aspect of disease risk prognostication, adapting interventions to stakeholder needs is critical to adoption. In usability testing, providers felt implementation would benefit complex patients and those with moderate risk of PAD based on traditional risk factors. These patients typically require extensive chart review and counseling. Thus, accurate models that can process complex patient histories and provide a summary risk score can be of high utility in these use cases. Furthermore, the cognitive load presented by such complex patients highlight the need for EHR integration and low-interaction interfaces to reduce further cognitive burden and navigation time^32^. In addition, clarifying the prediction score’s meaning and thresholds can facilitate usage by allowing providers to compare model output to their own internal schema. The next phase of implementation thus includes recruiting hospital information technology assistance to optimize EHR integration and exploring ways to clarify the prediction score. To this end, Norvell and colleagues report ways to present clarifying data in a clinical context. In a usability study of an amputation prediction tool, Norvell et al. used hover features, where clarifying text only appears when the pointer is hovering over an area. Such an approach can significantly reduce interface clutter^33^ and provide important model details at the point of care, potentially increasing likelihood of adoption.

We expected to find a large amount of skepticism towards use of machine learning-based models for risk assessment based on research that has previously reported barriers to acceptability of such approaches due to lack of model transparency and actionability^34^. However, the majority of practitioners we interviewed were receptive to the technology. For the minority of participants who cited concerns with machine learning models, proof of peer-review and increasing transparency regarding decision-making thresholds were mentioned as beneficial. Stakeholders also generated opportunities to increase actionability by identifying patients in whom model implementation could change their management. Even so, while physicians included in our study were generally receptive to utilizing the simulated dashboard, parallel interventions such as educational initiatives and identification of stakeholder champions will be needed to encourage real-world use.

Despite our promising results, a downside of employing machine learning, and especially deep learning methods, is that the lack of ample data can result in severe overfitting^35^. In the context of disease prediction, however, the widespread adoption of EHR data has made a large amount of patient data available^36^. To enable more widespread use our models will need to be validated prospectively, and ideally evaluated in multiple environments to get a better sense of real-world performance. Another area of growing concern is whether machine learning models can ultimately be fair, equitable, and impactful^37^. There is potential for EHR models to recapitulate detrimental biases in the healthcare system, such that those from disadvantaged health groups may continue to be disadvantaged with machine learning approaches. This is especially true for deep learning models where it remains difficult to identify which features the model may have weighted more heavily in making predictions. For instance, while risk factors in our traditional risk models were pre-defined, and we can extract from random forest-based models what features were most important to model predictions, we are not able to extract feature weights from our deep learning models. Future work will require assessment of how machine learning models perform in different groups prior to implementation and developing methods for interpretable feature extraction from deep learning models to ensure enable better evaluation of model metrics. Another limitation of our work is that we built our models through supervised learning which requires the laborious task of data labeling. Though we used the latest published techniques for PAD phenotyping^38,39^, given the nature of EHR data and the relatively low rates of PAD diagnosis, some patients may have been mislabeled. An alternative approach is to develop data sets in an unsupervised manner^40^. For example, many have proposed using machine learning and deep learning techniques to develop training data, which would decrease reliance on manual algorithms. Research is ongoing in evaluating how multiple metrics fare in comparison to using manually labeled data. Lastly, limitations to our usability testing approach include the artificial setting of the study and observer effect. Physicians interacted with the dashboard using remote videoconferencing, which enabled recording but may yield different results from a live patient encounter. Similarly, the presence of the observer may impact responses, although most of the observer speech was scripted in order to help standardize interactions.

In conclusion, we demonstrate the feasibility and performance of using machine learning and deep learning techniques coupled with EHR data to identify cases of PAD prior to diagnosis. We further detail the key components of implementation that need to be considered prior to model deployment. Future research will focus on prospective validation of our models and dashboard design for clinical use optimization.

## Supplementary Information

Supplementary Information.

## Data Availability

Given the sensitivity of the electronic health record data used, these data are not publicly available but are available to those working in direct collaboration with the corresponding author. All models were uploaded to GitHub for public use: https://github.com/SOM-RossLab/PAD-Detection-Models.
