# Supplementary material for: Performance and usability testing of an automated tool for detection of peripheral artery disease using electronic health records
Source: Sci Rep. 2022 Aug 3;12:13364. doi: 10.1038/s41598-022-17180-5 (PMC9349186; doi:10.1038/s41598-022-17180-5)
Supplement: Supplementary file 1 — Supplementary Information. [file 41598_2022_17180_MOESM1_ESM.pdf]

# Performance and usability testing of an automated tool for detection of peripheral artery disease using electronic health records

Ghanzouri, I[†1], Amal, S[†1], Ho, V[†1], Safarnejad, L[1], Cabot, J[1], Brown-Johnson, CG[2], Leeper, N[1,3], Asch, S[2], Shah, NH[3], Ross, EG*[1,3]

[1]Division of Vascular Surgery, Department of Surgery, Stanford University School of Medicine, Stanford, CA; [2]Primary Care and Population Health, Department of Medicine, Stanford, CA; [3]Center for Biomedical Informatics Research, Department of Medicine, Stanford University School of Medicine, Stanford, CA

# Supplemental materials

**Supplemental Table 1**. All codes used in definition of peripheral artery disease cohort.

| PAD Identification Codes | ICD-9 | ICD-10 | ICD-9-CM | ICD-10-PCS | CPT | Definition |
|---|---|---|---|---|---|---|
| Cases (codes used to include) | 440.2*, 440.3*, 440.8, 440.4, 443.9,<br><br>736.3*, 736.4*, 736.5, 736.6, 736.7*, 736.8*, 736.9, 735.*, 754.3*, 754.4*, 754.5*, 754.6*, 754.7*, 755.02, 755.13, 755.14, 755.3, 755.4, 755.6*, 755.8, 759.7, 759.89, 895.*, 896.*, 897.*, 820.*, 821.*, 822.*, 823.*, 824.*, 825.*, 826.*, 827.*, 828.*, 829.*, 835.*, 836.*, 837.*, 838.*, 904.*, 928.*, 929.*, 959.6, 959.7, 996.4*, 996.66, 996.67, 996.77, 996.78 | I70.2*, I70.3*, I70.4*, I70.5*, I70.6*, I70.7*, I70.8, I70.92, I73.9,<br><br>M21.05*, M21.06*, M21.07*, M21.15*, M21.16*, M21.17*, M21.7*, M21.869, M21.6X*, M21.85*, M21.86*, M21.95*, M21.96*, M20.1*, M20.2*, M20.3*, M20.*, M20.5*, M20.6*, Q65.**, Q68.1*, Q68.2*, Q68.3*, Q68.4*, Q68.5*, Q66.3*, Q66.4*, Q66.5*, Q66.6*, Q66.7*, Q66.8*, Q69.2*, Q70.2*, Q70.3*, Q72.**, Q74.8, Q74.9, | 39.50, 39.90, 38.18,<br><br>84.1* | 047C3*, 047D3*, 047H3*, 047J3*, 047K3*, 047L3*, 047M3*, 047N3*, 047P3*, 047Q3*, 047R3*, 047S3*, 047T3*, 047U3*, 047V3*, 047W3*, 047Y3*, 04C00ZZ, 04C03ZZ, 04CC0Z*, 04CC3Z*, 04CC4Z*, 04CD*, 04CH*, 04CJ*, 04CK*, 04CK*, 04CL*, 04CM*, 04CN*, 04CP*, 04CQ*, 04CR*, 04CS*, 04CT*, 04CU*, 04CV*, 04CY*, 04100J7, 04100J8, 04100JF, 04100JG, 04100JH, 04100JJ, 04100JK, 041C0JD, 041C0JH, 041D0JF, 041D0JJ,041H0JH, 041H0KJ, 041H4JH, 041J0JJ, 041J4JJ, 041K0J*, 041K0Z*, 041L09*, 041L0J*, 041L0K*, 041L0Z*, 041M*, 041N*, 041S0JQ, 041U0AP,<br><br>0Y6* | 37205, 37206, 37207, 37208, 37236, 37237, 37184, 37185, 37186, 35302, 35303, 35304, 35305, 35306, 35331, 35351, 35355, 35361, 35363, 35371, 35372, 35381, 35452, 35454, 35456, 35459, 35470, 35472, 35473, 35474, 35483, 35492, 35493, 35495, 35521, 35533, 35537, 35538, 35539, 35540, 35556, 35558, 35563, 35565, 35566, 35571, 35583, 35585, 35587, 35621, 35623, 35637, 35638, 35646, 35647, 35654, 35656, 35661, 35663, 35665, 35666, 35671, 35700, 35876, 35879, 35881, 35883, 35884, 37184, 37185, 37186, 37205, 37206, 37207, 37208, 0236T, 0237T, 0238T, 37225, 37224, 37227, 37226, 37222, 37223, 37220, 37221, 37229, 37228, 37231, 37230, 37233, 37232, | Defined as at least 2 mentions of a concept code (ICD-9/10, CPT, Observation) in a visit or note |

| | | | | | | |
|---|---|---|---|---|---|---|
| | | Q89.7, E78.71, E78.72, Q87.2, Q87.3, Q87.5, Q87.81, Q87.82, Q87.89, Q89.8, S72.**, S82.**, S92.**, T14.8*, M24.35*, M24.36*, M24.37*, S83.**, S93.**, S75.**, S79.**, S99.**, S77.**, S89.**, T84.** | | | 37235, 37234, 35548, 27590, 27591 ,27592, 27598, 27888, 27889, 28800, 28805 | |
| Controls (codes used to exclude) | 440.2*, 440.3*, 440.8, 440.4, 443.9 | I70.2*, I70.3*, I70.4*, I70.5*, I70.6*, I70.7*, I70.8, I70.92, I73.9 | 39.50, 39.90, 38.18, 84.1* | 047C3*, 047D3*, 047H3*, 047J3*, 047K3*, 047L3*, 047M3*, 047N3*, 047P3*, 047Q3*, 047R3*, 047S3*, 047T3*, 047U3*, 047V3*, 047W3*, 047Y3*, 04C00ZZ, 04C03ZZ, 04CC0Z*, 04CC3Z*, 04CC4Z*, 04CD*, 04CH*, 04CJ*, 04CK*, 04CK*, 04CL*, 04CM*, 04CN*, 04CP*, 04CQ*, 04CR*, 04CS*, 04CT*, 04CU*, 04CV*, 04CY*, 04100J7, 04100J8, 04100JF, 04100JG, 04100JH, 04100JJ, 04100JK, 041C0JD, 041C0JH, 041D0JF, 041D0JJ, 041H0JH, | 37205, 37206, 37207, 37208, 37236, 37237, 37184, 37185, 37186, 35302, 35303, 35304, 35305, 35306, 35331, 35351, 35355, 35361, 35363, 35371, 35372, 35381, 35452, 35454, 35456, 35459, 35470, 35472, 35473, 35474, 35483, 35492, 35493, 35495, 35521, 35533, 35537, 35538, 35539, 35540, 35556, 35558, 35563, 35565, 35566, 35571, 35583, 35585, 35587, 35621, 35623, 35637, 35638, 35646, | |

| | | | | | | |
|---|---|---|---|---|---|---|
| | | | | 041H0KJ, 041H4JH, 041J0JJ, 041J4JJ, 041K0J*, 041K0Z*, 041L09*, 041L0J*, 041L0K*, 041L0Z*, 041M*, 041N*, 041S0JQ, 041U0AP, 0Y6* | 35647, 35654, 35656, 35661, 35663, 35665, 35666, 35671, 35700, 35876, 35879, 35881, 35883, 35884, 37184, 37185, 37186, 37205, 37206, 37207, 37208, 0236T, 0237T, 0238T, 37225, 37224, 37227, 37226, 37222, 37223, 37220, 37221, 37229, 37228, 37231, 37230, 37233, 37232, 37235, 37234, 35548, 27590, 27591 ,27592, 27598, 27888, 27889, 28800, 28805 | |

ICD-9/ICD-10 – International Classification of Diseases, 9th Version/10th Version. ICD-9-CM International Classification of Diseases, 9th Version – Clinical Modification. ICD-10-PCS - International Classification of Diseases, 10th Version – Procedure Coding System.

**Supplemental Table 2. Codes used to define co-morbidities used in traditional risk factor modeling.**

| Disease | ICD-9 | ICD-10 | ICD-9-CM | ICD-10-PCS | CPT | Definition |
|---|---|---|---|---|---|---|
| Cerebrovascular disease | 434, 434, 434.01, 434.1, 434.9, 434.11, 434.91, 437, 437.1, 435, 435.1, 435.2, 435.3, 435.8, 435.9, 433, 433, 433.01, 433.1, 433.11, 433.2, 433.21, 433.21, 433.3, 433.8, 433.31, 433.81, 433.9, 433.91 | I66.01, I66.02, I66.03, I66.09, I66.11, I66.12, I66.13, I66.19, I66.21, I66.22, I66.23, I66.29, I66.3, I66.8, I63.30, I63.311, I63.312, | 38.12, 0.61, 39.28, 0.63 | 03CL***, 03CH***, 03CJ***, 03CK***, 03CM***, 03CN***, 03CP***, 03CQ***, 03CG***, 037H***, 037J***, 037K***, 037L***, 037M***, 037N***, 037H***, | 35301, 37215, 37216, 37195, 61645 | Defined as at least 2 mentions of a concept code (ICD-9/10, CPT, Observation) in a visit or note |

| | | | | | | |
|---|---|---|---|---|---|---|
| | | I63.313, I63.319, I63.321, I63.322, I63.323, I63.329, I63.331, I63.332, I63.333, I63.339, I63.341, I63.342, I63.343, I63.349, I63.39, I63.6, I66.9, I63.40, I63.411, I63.412, I63.413, I63.419, I63.421, I63.422, I63.423, I63.429, I63.431, I63.432, I63.433, I63.439, I63.441, I63.442, I63.443, I63.449, I63.49, I63.50, I63.511, I63.512, I63.513, I63.519, I63.521, I63.522, I63.523, I63.529, I63.531, I63.532, I63.533, I63.539, I63.541, I63.542, I63.543, I63.549, I63.59, I63.8, I63.9, I67.2, I67.82, G45.0, | | 037J***, 037K***, 037L***, 037M***, 037N*** | | |

| | | | | | | |
|---|---|---|---|---|---|---|
| | | G45.8, G45.1, G45.2, G45.9, I65.1, I63.22, I63.02, I63.22, I65.29, I65.21, I65.22, I65.23, I63.031, I63.032, I63.033, I63.039, I63.131, I63.132, I63.133, I63.139, I63.239, I65.09, I65.01, I65.02, I65.03, I63.219, I63.211, I63.212, I63.213, I63.011, I63.012, I63.013, I63.019, I63.111, I63.112, I63.113, I63.119, I65.8, I63.59, I63.09, I63.19, I63.29, I65.9, I63.20, I63.00, I63.10 | | | | |
| Coronary artery disease | 413, 413.1, 413.9, 411.1, 410, 410.01, 410.02, 410.1, 410.11, 410.12, 410.2, 410.21, 410.22, 410.4, 410.41, 410.42, | I20.8, I20.0, I21.01, I21.02, I21.09, I21.19, I21.11, I21.21, I21.29, | 36.01, 36.02, 36.03, 36.04, 36.05, 36.06, 36.07, 36.09, 0.66, | Z98.61, Z95.5, 0270***, 0271***, 0272***, 0273***, 02C0***, 02C1***, | 92920, 92921, 92924, 92925, 92928, 92929, 92933, 92934, | Defined as at least 2 mentions of a concept code (ICD9/10, CPT, Observation) |

| | | | | | | |
|---|---|---|---|---|---|---|
| | 410.3, 410.31, 410.32, 410.5, 410.51, 410.52, 410.6, 410.61, 410.62, 410.8, 410.81, 410.82, 410.7, 410.71, 410.72, 410.9, 410.91, 410.92, 412, 429.71, 429.79, 414, 414.01, 414.02, 414.03, 414.04, 414.05, 414.06, 414.07, 414.2, 414.3, 414.4, 414.8, 414.9 | I21.4, I21.3, I21.3, I22.**, I25.2, I23.1, I23.2, I23.0, I23.3, I23.4, I23.5, I23.6, I23.7, I23.8, I24.1, I25.10, I25.810, I25.811, I25.812, I25.110, I25.111, I25.118, I25.119, I25.700, I25.701, I25.708, I25.709, I25.710, I25.711, I25.718, I25.719, I25.720, I25.721, I25.728, I25.729, I25.730, I25.731, I25.738, I25.739, I25.750, I25.751, I25.758, I25.759, I25.760, I25.761, I25.768, I25.769, I25.790, I25.791, I25.798, I25.799, I25.82, I25.83, I25.84, I25.5, I25.89, I25.9 | V45.82, 36.1, 36.11, 36.12, 36.13, 36.14, 36.15, 36.16, 36.17, 36.18, 36.19, 36.2, V45.81 | 02C2***, 02C3***, 02703ZZ, 02704ZZ, 02713ZZ, 02714ZZ, 02723ZZ, 02724ZZ, 02733ZZ, 02734ZZ, 3E07017, 3E070PZ, 3E07317, 3E073PZ, Z95.1, 0210***, 0211***, 0212***, 0213*** | 92937, 92938, 92941, 92943, 92944, 92980, 92981, 92982, 92984, 92995, 92996, 92973, 92974, 33510, 33511, 33512, 33513, 33514, 33516, 33517, 33518, 33519, 33521, 33522, 33523, 33533, 33534, 33535, 33536 | |

| | | | | | | |
|---|---|---|---|---|---|---|
| Heart failure | 398.91, 402.01, 402.11, 402.91, 404.01, 404.03, 404.11, 404.13, 404.91, 404.93, 425.**, 428.** | I09.81, I11.0, I13.0, I13.2, I25.5, I42.*, I50.**, Z95.811 | 37.66, V43.21 | 02HA0QZ, 02HA4QZ, 02HA3QZ | 33979, 33982, 33983, 93750 | Defined as at least 2 mentions of a concept code (ICD9/10, CPT, Observation) |
| Hypertension | 401.**, 402.**, 403.**, 404.**, 405.** | I10.**, I11.**, I12.**, I13.**, I15.** | | | | Defined using at least 2 mentions of a concept code (ICD9/10, Observation) |
| Diabetes | 250.** | E10.**, E12.**, E13.**, E14.** | | | | Defined using at least 1 mention of a concept code (ICD9/10, Observation) AND on at least 1 glycemic medication OR Hemaglobin A1c (hgbA1c) > 6.4 |
| Hyperlipidemia | 272.0*, 272.1*, 272.2*, 272.4* | E78.0*, E78.1*, E78.2*, E78.4*, E78.5* | | | | Defined using at least 1 mention of a concept code (ICD9/10, Observation) AND on at least 1 lipid medication OR cholesterol > = 200 OR LDL >= 100 OR triglycerides >= 150 |
| Obesity | 278.00, 278.01 | E66.0 , E66.01, E66.09, E66.9 | | | | Defined using at least 2 mentions of a concept code (ICD9/10, Observation) OR a body mass index measurement >= 30 |
| Current smoker | | | 305.1 | F17.210, F12.213, F17.218, F17.219, | | At least 2 codes during their care |

| | | | | | | |
|---|---|---|---|---|---|---|
| | | | | O99.330, O99.331, O99.332, O99.333, O99.334, O99.335, T65.221, T65.222, T65.223, T65.224, Z71.6, Z72.0 | | |

ICD-9/ICD-10 – International Classification of Diseases, 9th Version/10th Version. ICD-9-CM International Classification of Diseases, 9th Version – Clinical Modification. ICD-10-PCS - International Classification of Diseases, 10th Version – Procedure Coding System.

**Supplemental Table 3.** Logistic regression model performance for outer 5-folds.

| | AUC | Specificity | Sensitivity | Standard Dev. | 95% CI |
|---|---|---|---|---|---|
| **Fold 1** | 0.807 | 0.736 | 0.73 | 0.0108 | 0.786-0.818 |
| **Fold 2** | 0.798 | 0.678 | 0.774 | 0.0105 | 0.778-0.822 |
| **Fold 3** | 0.819 | 0.742 | 0.751 | 0.0103 | 0.798-0.839 |
| **Fold 4** | 0.826 | 0.712 | 0.792 | 0.0099 | 0.807-0.846 |
| **Fold 5** | 0.802 | 0.782 | 0.680 | 0.0103 | 0.782-0.822 |
| **Average** | 0.810 | 0.730 | 0.746 | | |

AUC – area under the curve

**Supplemental Table 4.** Nomogram model performance for outer 5-folds.

| | AUC | Specificity | Sensitivity | Standard Dev. | 95% CI |
|---|---|---|---|---|---|
| **Fold 1** | 0.641 | 0.708 | 0.558 | 0.0270 | 0.588-0.694 |
| **Fold 2** | 0.616 | 0.551 | 0.603 | 0.0279 | 0.567-0.671 |
| **Fold 3** | 0.633 | 0.538 | 0.644 | 0.0280 | 0.579-0.688 |
| **Fold 4** | 0.658 | 0.598 | 0.680 | 0.0276 | 0.604-0.712 |
| **Fold 5** | 0.660 | 0.688 | 0.610 | 0.0272 | 0.607-0.713 |
| **Average** | 0.642 | 0.617 | 0.619 | | |

Nomogram scores are calculated based on patient demographic features in accordance to Duval et al (2012). AUC – area under the curve.

**Supplemental Table 5.** Machine learning model performance for outer 5-folds using random forest architecture.

| | AUC | Specificity | Sensitivity | Standard Dev. | 95% CI |
|---|---|---|---|---|---|

| | | | | | |
|---|---|---|---|---|---|
| **Fold 1** | 0.908 | 0.797 | 0.845 | 0.0061 | 0.896-0.919 |
| **Fold 2** | 0.904 | 0.802 | 0.819 | 0.0061 | 0.892-0.916 |
| **Fold 3** | 0.911 | 0.832 | 0.817 | 0.0063 | 0.898-0.923 |
| **Fold 4** | 0.909 | 0.815 | 0.836 | 0.0062 | 0.897-0.921 |
| **Fold 5** | 0.914 | 0.792 | 0.852 | 0.0056 | 0.902-0.925 |
| **Average** | 0.909 | 0.808 | 0.834 | | |

AUC – area under the curve; CI – Confidence interval.

**Supplemental Table 6**. Deep learning model performance for outer 5-folds.

| | **AUC** | **Specificity** | **Sensitivity** | **Standard Dev.** | **95% CI** |
|---|---|---|---|---|---|
| **Fold 1** | 0.968 | 0.895 | 0.944 | 0.0018 | 0.959-0.976 |
| **Fold 2** | 0.963 | 0.860 | 0.970 | 0.0016 | 0.954-0.972 |
| **Fold 3** | 0.956 | 0.869 | 0.922 | 0.0023 | 0.946-0.966 |
| **Fold 4** | 0.956 | 0.885 | 0.944 | 0.0022 | 0.945-0.967 |
| **Fold 5** | 0.974 | 0.868 | 0.970 | 0.0016 | 0.967-0.981 |
| **Average** | 0.963 | 0.875 | 0.950 | | |

AUC – area under the curve; CI – Confidence interval.
